# A biology-driven approach identifies the hypoxia gene signature as a predictor of the outcome of neuroblastoma patients

**DOI:** 10.1186/1476-4598-9-185

**Published:** 2010-07-12

**Authors:** Paolo Fardin, Annalisa Barla, Sofia Mosci, Lorenzo Rosasco, Alessandro Verri, Rogier Versteeg, Huib N Caron, Jan J Molenaar, Ingrid Øra, Alessandra Eva, Maura Puppo, Luigi Varesio

**Affiliations:** 1 Laboratory of Molecular Biology, Gaslini Institute, Genoa, Italy; 2 Department of Computer and Information Science, University of Genoa, Genoa, Italy; 3 Physics Department, University of Genoa, Genoa, Italy; 4 Center for Biological & Computational Learning, MIT, Cambridge, MA, USA; 5 Department of Human Genetics, Academic Medical Center, University of Amsterdam, Amsterdam, the Netherlands; 6 Department of Pediatric Oncology, Academic Medical Center, University of Amsterdam, the Netherlands

## Abstract

**Background:**

Hypoxia is a condition of low oxygen tension occurring in the tumor microenvironment and it is related to poor prognosis in human cancer. To examine the relationship between hypoxia and neuroblastoma, we generated and tested an in vitro derived hypoxia gene signature for its ability to predict patients' outcome.

**Results:**

We obtained the gene expression profile of 11 hypoxic neuroblastoma cell lines and we derived a robust 62 probesets signature (NB-hypo) taking advantage of the strong discriminating power of the *l*_1_-*l*_2 _feature selection technique combined with the analysis of differential gene expression. We profiled gene expression of the tumors of 88 neuroblastoma patients and divided them according to the NB-hypo expression values by K-means clustering. The NB-hypo successfully stratifies the neuroblastoma patients into good and poor prognosis groups. Multivariate Cox analysis revealed that the NB-hypo is a significant independent predictor after controlling for commonly used risk factors including the amplification of *MYCN *oncogene. NB-hypo increases the resolution of the *MYCN *stratification by dividing patients with *MYCN *not amplified tumors in good and poor outcome suggesting that hypoxia is associated with the aggressiveness of neuroblastoma tumor independently from *MYCN *amplification.

**Conclusions:**

Our results demonstrate that the NB-hypo is a novel and independent prognostic factor for neuroblastoma and support the view that hypoxia is negatively correlated with tumors' outcome. We show the power of the biology-driven approach in defining hypoxia as a critical molecular program in neuroblastoma and the potential for improvement in the current criteria for risk stratification.

## Background

Neuroblastoma is the most common pediatric solid tumor, deriving from ganglionic lineage precursors of the sympathetic nervous system [1,2]. It is diagnosed during infancy and shows notable heterogeneity with regard to histology and clinical behavior [3], ranging from rapid progression associated with metastatic spread and poor clinical outcome to spontaneous, or therapy-induced regression into benign ganglioneuroma [4]. Clinical and molecular risk factors which correlate with prognosis include age at diagnosis, stage, histology, chromosomal aberrations, and amplification of the N-myc proto-oncogene (*MYCN*), which is the most typical genetic feature of advanced-stage neuroblastoma [3,5,6]. *MYCN *amplification correlates with a more malignant course of the disease, angiogenesis, resistance to therapy, and poor clinical outcome [5,7-9], suggesting that it may be a progression-related event and a potential therapeutic target [3].

In particular, high expression of the proangiogenic cytokine, vascular endothelial growth factor (VEGF), is a marker of poor prognosis in neuroblastoma tumors [10,11].

Tumor microenvironment is intimately connected with the evolution of the disease. In particular, hypoxia, a condition of low oxygen tension occurring in poorly vascularized areas, has a profound effects on tumor cell growth, susceptibility to apoptosis, and resistance to radio- and chemotherapy [12,13]. The response to hypoxia is associated with changes in gene expression [14-16]. Hypoxia activates, among others, hypoxia-inducible transcription factors (HIF-1α and HIF-2α) [12,17,18], which transactivate the hypoxia-responsive element (HRE) present in the promoter of many genes encoding angiogenic, metabolic and metastatic factors [13,19,20] and contribute to the acquisition of the tumor aggressive phenotype [13,14,21].

There is little information on the relationship among hypoxia, tumor phenotypes and clinical parameters in neuroblastoma. Rapidly expanding neuroblastoma tumors present hypoxic areas and metastasize to bone marrow [22] and it has been reported that HIF-2α is significantly correlated with a poor prognosis [23]. Neuroblastoma cells adaptation to hypoxia activates a gene expression program consistent with the pro-metastatic events [24]. Furthermore, hypoxia causes dedifferentiation in vitro and in vivo suggesting a novel mechanism for the selection of highly malignant neuroblastoma cells with stem-cell characteristics [25].

Every cell type is bound to respond to hypoxia and the gene expression profile of the tumor will have, to some extent, the footprint of hypoxia which, in turn, might be a prognostic indicator depending on the proportion of the hypoxic tissue. Therefore, clues to the prognosis of neuroblastoma might be reflected at the time of surgical removal in the pattern of hypoxic gene expression in the primary tumor. Definition, detection and analysis of hypoxia induced gene expression have the potential of leading to interesting and useful molecular predictors of neuroblastoma progression.

We investigated the prognostic potential of hypoxia induced genes in neuroblastoma tumors. A biology-driven approach was chosen to define the hypoxia signature. We performed a systematic analysis of the transcriptome of neuroblastoma cell lines cultured under hypoxic or normoxic conditions and applied a rigorous framework to derive a robust 62 probesets neuroblastoma hypoxia signature (NB-hypo). We show that the NB-hypo have a strong predictive power in the multivariate Cox regression model that includes the classical prognostic factors. This signature can also stratify a heterogeneous subgroup of patients with *MYCN *not amplified tumors. In conclusion, we demonstrate that NB-hypo is an independent risk factor and provide evidences of the power of the biology-driven approach to study the role of molecular programs in human tumors.

## Materials and methods

### Patients

The clinical characteristics of the 88 patients used in this study are listed in Table 1. The tumor samples were obtained at the time of diagnosis. The median follow-up time for patients in this study is 3.5 years (range = 0-16 years) and the median age at the diagnosis is 1 year (range = 0-14). Analysis of the predictive value of the current European risk factors *MYCN *status, International Neuroblastoma Staging System (INSS), and age was performed by Kaplan-Meier curves and log-rank test for overall survival (OS). All the prognostic factors stratified the patients in our cohort (p < 0.0001). Overall survival was 72.6% for the *MYCN *not amplified patients, compared with 10.4% for the *MYCN *amplified patients (HR = 4.30, 95% CI 3.66-30.69). For INSS, OS curves were characterized by a survival rate of 93.6% for the stage 1, 2, 3, and 4S patients, compared with a survival rate of 25.4% for the stage 4 patients (HR = 16.65, 95% CI 5.37-23.74). Overall survival was 100% for the patients with age at the diagnosis < 1 year, compared with 46.6% for the patients with age at the diagnosis > 1 year (HR and 95% CI undefined).

**Table 1:** Clinical characteristics of 88 neuroblastoma patients

	number	%of total
**age**		
< 1	25	28.4
> 1	63	71.6
		

**INSS stage**		
1	8	9.1
2	15	17.0
3	13	14.8
4	40	45.5
4s	12	13.6
		

***MYCN *status**		
Normal	72	81.8
Amplified	16	18.2
		

**tissue source**		
adrenal gland	34	38.6
liver	8	9.1
lymphnodes	8	9.1
side chain abd	20	22.7
side chain thorax	10	11.4
skin	2	2.3
Undetermined	6	6.8

### Cell lines and culture conditions

The human neuroblastoma cell lines GI-LI-N, ACN, GI-ME-N, IMR-32, LAN-1, SK-N-BE(2)C, SK-N-F1, and SK-N-SH were purchased from the Interlab Cell Line Collection while SHEP-2, SHEP-21N over-expressing MYCN, and SHEP-21N not over-expressing MYCN were kindly provided by Dr. M. Schwab (Division of Tumour Genetics, German Cancer Research Centre, Heidelberg, Germany). The cell lines were cultured in RPMI 1640 (Euroclone Ltd., Celbio, Milan, Italy), supplemented with 10% heat-inactivated fetal bovine serum (Sigma, Milan Italy), 2 mmol/L L-glutamine, 10 mM Hepes, 100 units/mL penicillin, and 100 μg/mL streptomycin (Euroclone Ltd), at 37°C in a humidified incubator containing 20% O2, 5% CO2, and 75% N2. Hypoxic conditions (1% O2) were achieved by culturing the cells in an anaerobic workstation incubator (BUG BOX, Jouan, ALC International S.r.l., Cologno Monzese, Milan, Italy) flushed with a gas mixture containing 1% O2, 5% CO2, and balanced N2 at 37°C in a humidified atmosphere. Oxygen tension in the medium was measured with a portable, trace oxygen analyzer (Oxi 315i/set, WTW; VWR International, Milan, Italy).

### RNA extraction and microarray experiments

Total RNA was extracted using Trizol (Invitrogen Life technologies, Irvine, CA) according to the manufacturer's instructions. RNA was resuspended in diethyl pyrocarbonate-treated H2O (DEPC water), the physical quality control of RNA integrity was carried out by electrophoresis using Agilent Bioanalyzer 2100 (Agilent Technologies Waldbronn, Germany) and quantified by NanoDrop (NanoDrop Technologies Wilmington, Delawere USA). Total RNA was reverse transcribed into cDNA and biotin labeled according to the Affymetrix instructions (Affymetrix, SantaClara, CA). Biotin-labeled cRNA was cleaned up with the Qiagen RNeasy Mini kit and ethanol precipitation, checked for quality with Agilent Bioanalyzer 2100, and fragmented by incubation at 94°C for 35 min in 40 mmol/L Tris-acetate (pH 8.1), 100 mmol/L potassium acetate, and 30 mmol/L magnesium acetate. Fragmented cRNA was used for hybridization to Affymetrix HG-U133 Plus 2.0 arrays. GeneChips were scanned using an Affymetrix GeneChip Scanner 3000. The microarray data were released into the GEO-database (accession number GSE17714). Expression values were quantified, and array quality control was performed using the statistical algorithms implemented in Affymetrix Microarray Suite 5.0. All microarrays were examined for surface defects, grid placement, and background intensity. All the global microarray quality metrics that are summarized in Affymetrix report files were within normal ranges for all the samples. The scale factors (SF) for all the hybridizations were within 1 SD of the mean (SF 1-3). All samples had 3'/5' *Gapdh *and 3'/5' *Actb *ratios below the maximum threshold of 3. To asses RNA integrity, "RNA digestion plot" was performed. Quality control and RNA digestion plot were used as implemented in the R package affy. Array signals were scaled to an average intensity of 500, and the resulting data were normalized as follows: 1) per microarray sample, dividing the raw data by 50th percentile of all measurements; and 2) per gene, by dividing the normalized data by the median of the expression level for the gene in all samples.

### Data analysis and statistics of gene expression in cell lines

To define the hypoxia gene signature, we independently applied the *l*_1_-*l*_2 _regularization with double optimization and the evaluation of differential gene expression to the dataset of 11 neuroblastoma cell lines cultured in hypoxic or normoxic conditions. The signature was defined as the intersection of the probesets selected by the two methods.

*l*_1_-*l*_2 _regularization technique was proposed in the context of statistical learning as a feature selection method [26] and successfully applied in the field of computational biology [27]. *l*_1_-*l*_2 _regularization is a supervised multivariate analysis which statistical significance and model selection is performed within double selection bias free cross-validation loops. Detailed descriptions of the application of *l*_1_-*l*_2 _algorithm and the method applied to select optimal values of the parameters for signature definition were previously reported [28]. Briefly, the output of the *l*_1_-*l*_2 _regularization algorithm depends on one free parameter εthat governs the amount of correlation allowed among the selected variables (probesets); the higher the ε, the more probesets are taken into account. The algorithm analyzed all the probesets on the chip simultaneously, thereby dealing with 54675-dim vectors. The system was characterized by a leave-one-out error of 10% and performed the validation loop producing 22 lists of probesets for each εvalue. A common list was obtained as the union of the 22 lists, with a frequency score counting how many times each probeset was selected by the algorithm in the 22 cross validation loops. In order to define the frequency threshold, the behavior of each ε curve was analyzed. The minimal list is obtained for values of ε equal to or lower than 1, whereas the largest list, which is correlation aware, is obtained for ε equal to 100. Thus, to include every probesets concurring in the identification of the hypoxia status, we set ε equal to 100.

In order to define the differentially expressed probesets, a fold change was calculated as the ratio between the expression level in the hypoxic and normoxic conditions for each individual cell line. The probesets that were modulated of at least 2-fold were selected and a t-test with Benjamini and Hochberg multiple hypothesis correction was applied. Only those probesets that were selected by both *l*_1_-*l*_2 _regularization algorithm and differentially expressed method were included in the NB-hypo.

### Data analysis and statistics of gene expression in neuroblastoma tumors

The predictive value of the signature was assessed on the tumor expression dataset. The patients were divided into two clusters by applying K-means clustering, with euclidean distance and 100 iterations, on the normalized expression values of the NB-hypo.

We performed a t-test to assess the significance of the obtained clustering versus the random clustering distribution. A permutation test was performed in order to measure the stability of the obtained clusters. We sampled 300 times a random signature and repeated the clustering procedure, each time evaluating the misclassification distance between the obtained and random clusters. Hierarchical clustering analysis with complete linkage was performed by Cluster 3.0 software (Michael Eisen, Stanford University, California, USA). The dendrogram was visualized using TreeView 1.6 (Michael Eisen, Stanford University, California, USA).

The probability of overall survival (OS) and event-free survival (EFS) was calculated using Kaplan-Meier method, and the significance of the difference between Kaplan-Meier curves was calculated by the log-rank test using Prism 4.03 (GraphPad Software, Inc.).

Multivariate Cox proportional regression analysis was performed to evaluate the prognostic significance of NB-hypo and the currently used risk factors such as age at diagnosis (> 1 year *vs*. < 1 year), International Neuroblastoma Staging System (INSS) stage (stage 4 *vs*. not stage 4), and *MYCN *status (amplified *vs*. not amplified). Hazard ratios and 95% confidence interval for survival were calculated (StatPlus 4.7, AnalystSoft).

## Results

### The hypoxia signature of neuroblastoma cell lines

**Figure 1:**
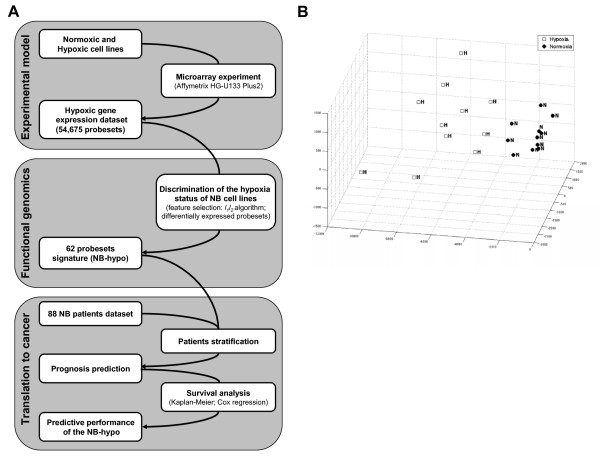
**Definition of neuroblastoma hypoxia signature (NB-hypo)**. A, workflow of the biology-driven approach applied to define the hypoxia signature. The workflow is divided into three main blocks representing, from top to bottom, the experimental data collection, the functional genomics data analysis, and the evaluation of the NB-hypo as prognostic factor in neuroblastoma patients. B, principal components representation of the multivariate analysis performed on the 11 cell lines by *l*_1 _- *l*_2 _algorithm. This figure illustrates a 3-dimensional visualization of the dataset restricted to the selected probesets projected on their 3 principal components. Open squares (H) represent the cell lines in hypoxic status and the black circles (N) the corresponding cell lines in normoxic status.

The neuroblastoma hypoxia signature was defined by the biology-driven approach outlined in Figure 1A. We utilized an in vitro experimental model consisting of 11 neuroblastoma cell lines, cultured under normoxic or hypoxic conditions for 18 hrs. RNA was extracted, processed and the gene expression profile was determined. To derive the hypoxia signature we applied the framework detailed in the Materials and Methods' section, which combines the discriminatory power of the *l*_1 _- *l*_2 _regularization algorithm and the biological strength of differentially expressed genes. The application of *l*_1 _- *l*_2 _algorithm to the neuroblastoma cell lines dataset defined a list of 137 probesets able to discriminate hypoxic from normoxic cell lines (Figure 1B). In the figure the multi dimensional model is projected on its 3 principal components. The differential expression analysis of hypoxic vs. normoxic cell lines identified 174 significant modulated probesets. We intersected the cluster of probesets originated from the *l*_1 _- *l*_2 _regularization with that created by the analysis of the differential expression, obtaining a 62 probesets signature that is referred to as NB-hypo. The 62 probesets correspond to 32 genes (Table 2) comprising mainly known hypoxia inducible genes providing indirect validation of our signature. The NB-hypo, which was generated only by objective criteria, was then applied to the gene expression profiles of tumors from neuroblastoma patients.

**Table 2:** NB-hypo genes

Gene Name	GenBank*	Description	Reference^†^
***AK3L1***	NM_013410	adenylate kinase 3-like 1	[14]
*ALDOC*	NM_005165	aldolase C, fructose-bisphosphate	[71]
***ANGPTL4***	NM_016109	angiopoietin-like 4	[72]
***ANKRD37***	AA886870	ankyrin repeat domain 37	[73]
*BHLHB2*	NM_003670	basic helix-loop-helix domain containing, class B, 2	[74]
***BNIP3***	NM_004052	BCL2/adenovirus E1B 19 kDa interacting protein 3	[43]
*BNIP3L*	AF060922	BCL2/adenovirus E1B 19 kDa interacting protein 3-like	[75]
*BTG1*	NM_001731	B-cell translocation gene 1, anti-proliferative	[76]
***DDIT4***	NM_019058	DNA-damage-inducible transcript 4	[43]
*E2IG5*	NM_014367	growth and transformation-dependent protein	[77]
*EGLN1*	BC005369	egl nine homolog 1 (C. elegans)	[78]
*EGLN3*	NM_022073	egl nine homolog 3 (C. elegans)	[79]
*FUT11*	BF541967	fucosyltransferase 11 (alpha (1,3) fucosyltransferase)	--
***IGF1R***	H05812	insulin-like growth factor 1 receptor	[80]
*IGFBP3*	M31159	insulin-like growth factor binding protein 3	[81]
*JMJD1A*	AA524505	jumonji domain containing 1A	[82]
*MAPT*	AI056359	microtubule-associated protein tau	--
*MTP18*	AF060924	mitochondrial protein 18 kDa	--
*MXI1*	NM_005962	MAX interactor 1; MAX interactor 1	[83]
***NDRG1***	NM_006096	N-myc downstream regulated gene 1	[84]
*P4HA2*	NM_004199	procollagen-proline, 2-oxoglutarate 4-dioxygenase, alpha polypeptide II	[85]
*PDK1*	NM_002610	pyruvate dehydrogenase kinase, isoenzyme 1	[71]
***PFKFB4***	AL038787	6-phosphofructo-2-kinase/fructose-2,6-biphosphatase 4	[86]
***PGK1***	NM_000291	phosphoglycerate kinase 1	[87]
*PGM1*	NM_002633	phosphoglucomutase 1	[88]
***PLOD1***	NM_000302	procollagen-lysine 1, 2-oxoglutarate 5-dioxygenase 1	[85]
*SLC2A3*	NM_006931	solute carrier family 2 (facilitated glucose transporter), member 3	[89]
***TNIP1***	NM_006058	TNFAIP3 interacting protein 1	--
*TPI1*	NM_000365	triosephosphate isomerase 1	[90]
*TXNIP*	AA812232	thioredoxin interacting protein	[81]
***VEGF***	AF022375	vascular endothelial growth factor	[71]
*ZNF395*	NM_017606	zinc finger protein 395	[91]

* GenBank accession number.

^† ^Representative references for genes previously shown to be modulated by hypoxia.

The genes that overlap with other hypoxia signatures are in bold.

### Discriminating power of the NB-hypo

**Figure 2:**
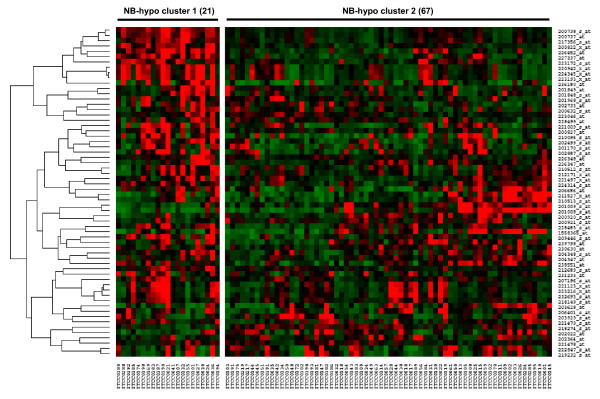
**Heatmap of the 62 probesets in the 88 neuroblastoma tumors**. The expression data for each individual probeset have been scaled and are represented by pseudo-colors in the heatmap. Red color corresponds to high level of expression and green color corresponds to low level of expression. The 88 patients (columns) were divided into two groups by k-means clustering. Cluster 1 consists of 21 patients and cluster 2 consists of 67 patients. The expression values of the 62 probesets were grouped by hierarchical clustering (rows). Hierarchical clustering dendrogram is on the left and the corresponding probesets on the right.

To study the prognostic value of the NB-hypo, we classified 88 neuroblastoma patients into two groups by applying a k-means clustering to the gene expression values of the 62 probesets (Figure 2). The significance of the clustering performance was assessed by permutation tests of 62 random probesets. The misclassification distance was calculated each time and the t-test (p < 0.001, data not shown) indicated that the NB-hypo significantly stratified patients into two clusters of 21 (cluster 1) and 67 (cluster 2) patients respectively. The expression levels of the probesets were grouped by hierarchical clustering and are represented in the heatmap in Figure 2. Cluster 1 consists of tumors in which the hypoxia probesets are highly expressed and stable whereas cluster 2 consists of tumors in which the expression levels are lower and less stable.

**Figure 3:**
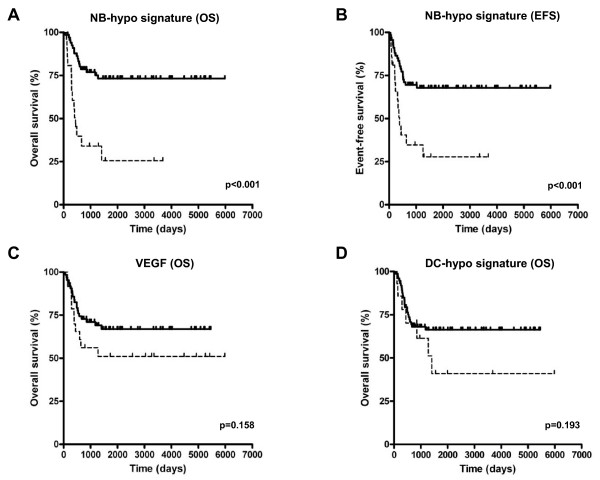
**Kaplan-Meier and log-rank analysis for 88 neuroblastoma patients**. OS (A) and EFS (B) of patients classified according to the NB-hypo. OS (C) of patients classified according to 4 *VEGF *probesets. OS (D) of patients classified according to dendritic cells hypoxia signature. Solid and dashed curves represent good and poor prognosis patients, respectively. The p-value of the log-rank test is shown.

The survival in the resulting groups was analyzed by Kaplan-Meier curves, log-rank test for overall survival (OS) (Figure 3A) and event-free survival (EFS) (Figure 3B). The results show a significant separation (p < 0.001) of the two curves in both OS and EFS demonstrating that the NB-hypo can stratify patients into poor (cluster 1) and good prognosis (cluster 2) groups. OS curves are characterized by a survival rate of 73.2% for the patients with good prognosis, compared with a survival rate of 25.5% for the patients with poor prognosis (HR = 4.26, 95% CI 3.33-22.81). EFS curves are characterized by a survival rate of 67.7% for the patients with good prognosis, compared with a survival rate of 27.7% for the patients with poor prognosis (HR = 3.08, 95% CI 1.94-11.09). The latter belong to the class with highly expressed hypoxia-related probesets (Figure 2), suggesting that the hypoxic microenvironment takes part in the definition of the neuroblastoma aggressiveness.

As a control, we tested the discriminating power of the four probesets characterizing *VEGF*, a gene represented in the NB-hypo and broadly up-regulated in hypoxic cells. The expression values of the four probesets were used to classify the 88 patients into two groups by k-means clustering (Figure 3C). *VEGF *alone was not capable of dividing the patients in statistically significant groups demonstrating the need for a complex signature.

To address the question of the relationship between effectiveness of the hypoxia signature and cell lineage, we tested the hypoxia signature of human dendritic cells [29]. The latter signature did not stratify the patients (Figure 3D) indicating that the prognostic value of the NB-hypo was dependent on the nature of the cell lines used for its definition.

To determine whether the NB-hypo is an independent risk predictor over the currently used risk factors, we created a multivariate Cox proportional hazard regression model using NB-hypo, age, INSS stage, and *MYCN *status. The results are shown in Table 3. INSS stage and NB-hypo have been found to be significant predictors (p < 0.01), with Hazard Ratios (HR) of 7.15 (95% CI, 2.08 to 24.64) and 2.73 (95% CI, 1.31 to 5.69) respectively, thus providing the evidence that hypoxia is a prognostic factor in neuroblastoma patients. There is no a significant correlation between INSS stage and NB-hypo. However, every stage 2 and stage 3 patients who survived and every stage 1 patients were classified by NB-hypo in the good prognosis group, while in the poor prognosis group 13 out of 21 (62%) were stage 4 patients (data not shown).

**Table 3:** Multivariate Cox analysis for 88 patients

Multivariate Cox regression				
**Factor**	***P***	**HR**	**95% CI**	

			**Lower**	**Upper**

				
INSS Stage (1-3, 4S vs 4)	**0.002**	7.15	2.08	24.64
NB-hypo	**0.007**	2.73	1.31	5.69
Age (< 1y vs > 1y)	0.801			
*MYCN *(normal vs amplified)	0.671			

We studied the relationship between NB-hypo and other hypoxia signatures generated in different tumor systems in terms of overlapping, neuroblastoma patients' stratification and risk assessment. We considered the following hypoxia signatures: the hypoxia metagene described by Winter *et al*. [30] and redefined by Buffa *et al*. [31]; the VEGF signature by Hu *et al*. [32]; two early hypoxia signatures by Seigneuric *et al*. [33]. First, we investigated whether the 32 genes composing our signature are part of the considered hypoxia signatures. Table 4 shows the result of the overlap analysis. Few genes belonging to the NB-hypo are found in each of the other hypoxia signatures. Moreover, a total of 12 out of 32 genes are represented in at least one of the other signatures (genes in bold in Table 2). We then tested the predictive performance of the hypoxia signatures in stratifying neuroblastoma patients by performing survival curves analysis and multivariate Cox regression. The results are reported in Table 5 and demonstrate that most of these signatures fail to stratify the patients, with the exception of Winter's hypoxia signature. However it is not a significant predictor in the multivariate Cox analysis. These results demonstrate the effectiveness of the biology-driven approach applied to derive the neuroblastoma hypoxia signature and that hypoxia signatures derived from different tumor types are less effective than the one derived from the same tumor type for which they are conceived. In conclusion, we show that the NB-hypo is an independent and significant risk factor that can predict neuroblastoma patients' outcome.

**Table 4:** Hypoxia gene signatures overlapping

	Number of genes	Overlapping genes*
Hypoxia metagene(Winter) [30]	99	7
Hypoxia metagene(Buffa) [31]	51	8
VEGF signature [32]	13	5
Early hypoxia signature 0% [33]	71	1
Early hypoxia signature 2%[33]	34	0

* Number of overlapping genes among NB-hypo and the other signatures.

**Table 5:** Prognostic significance of other hypoxia signatures compared to NB-hypo

	**Hypoxia metagene (Winter) **[30]	**Hypoxia metagene (Buffa) **[31]	**VEGF signature **[32]	**Early hypoxia signature 0% **[33]	**Early hypoxia signature 2% **[33]	NB-hypo
log-rank test*	**0.006**	0.945	0.891	0.225	0.414	**< 0.001**
P^†^	0.248	0.827	0.649	0.012	0.636	**0.007**
HR	1.57	1.11	1.25	6.35	1.22	2.73
95% CI	(0.73, 3.35)	(0.44, 2.75)	(0.47, 3.31)	(1.50, 27.03)	(0.54, 2.76)	(1.31, 5.69)

* p-value of the log-rank test for overall survival patients stratification.

^† ^p-value for the multivariate Cox analysis for 88 patients.

Significant p-values (p < 0.01) are in bold.

### NB-hypo as a prognostic factor in MYCN not amplified patients

**Figure 4:**
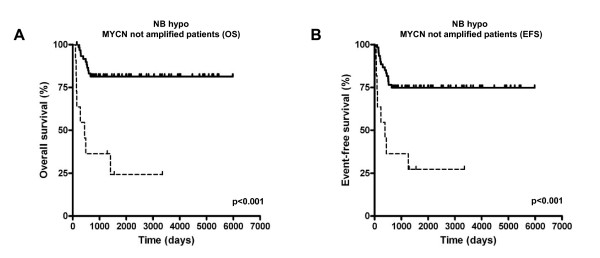
**Kaplan-Meier and log-rank analysis for 72 neuroblastoma patients without *MYCN *amplification**. OS (A) and EFS (B) of patients classified according to the NB-hypo. Solid and dashed curves represent good and poor prognosis patients, respectively. The p-value of the log-rank test is shown.

*MYCN *amplification is a very important risk factor in neuroblastoma and correlates with unfavorable prognosis. However, within patients lacking *MYCN *amplification, the commonly used prognostic factors give little information regarding outcome [34]. Thus, it was important to determine the predictive power of the NB-hypo in *MYCN *not amplified tumors found in 72 out of 88 patients. We classified the neuroblastoma patients into two classes by applying k-means clustering to the gene expression values of the 62 probesets. The Kaplan-Meier curves and log-rank test for overall survival (OS) (Figure 4A) and event-free survival (EFS) (Figure 4B) are shown. The results demonstrate a significant separation of the two curves in both OS (p < 0.001) and EFS (p < 0.001) thereby identifying good and poor prognosis *MYCN *not amplified patients. The good prognosis cluster consists of 61 patients, whereas 11 patients belong to the poor prognostic cluster. OS curves are characterized by a survival rate of 81.4% for the patients with good prognosis, compared with a survival rate of 24.2% for the patients with poor prognosis (HR = 6.71, 95% CI 8.74-182.30). EFS curves are characterized by a survival rate of 74.8% for the patients with good prognosis, compared with a survival rate of 27.3% for the patients with poor prognosis (HR = 4.53, 95% CI 3.49-50.25).

**Figure 5:**
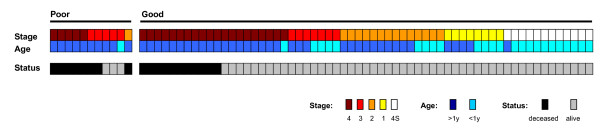
**Risk factors and survival of 72 *MYCN *not amplified patients**. Patients are divided in poor and good prognosis groups according to the NB-hypo. Columns represent individual patients. The first line represents the patients according to the International Neuroblastoma Staging System (INSS). The second line represents the patients according to the age at diagnosis (> 1 year *vs*. < 1 year). In the last line, the patients are divided in deceased, black squares, and alive, gray squares.

We created a multivariate Cox proportional hazard regression model of age, INSS stage and NB-hypo (Table 6). The NB-hypo (p = 0.001; HR = 5.04; 95% CI, 2.00 to 12.69) is a significant independent predictor of outcome and it is equivalent to the INSS stage (p = 0.002; HR = 7.35; 95% CI, 2.13 to 25.40). Although the limited number of patients did not allow further sub grouping with statistical significance, it is interesting to analyze the association of the patients' risk factors profile with poor and good prognosis groups (Figure 5). All stage 1 and stage 4S patients and every stage 2 and stage 3 patients who survived were correctly classified by NB-hypo as good prognosis. Interestingly, NB-hypo correctly classified in the poor prognosis group the only stage 3, age>1 year intermediate risk and stage 2, age>1 year low risk patients who died.

**Table 6:** Multivariate Cox analysis for 72 patients without *MYCN *amplification

Multivariate Cox Regression				
**Factor**	***P***	**HR**	**95% CI**	

			**Lower**	**Upper**

				
INSS Stage (1-3, 4S vs 4)	**0.002**	7.35	2.13	25.40
NB-hypo	**0.001**	5.04	2.00	12.69
Age (< 1y vs > 1y)	0.868			

Our results demonstrate that NB-hypo increases the resolution of the *MYCN *stratification by dividing patients with *MYCN *not amplified tumors in good and poor outcome groups suggesting that the neuroblastoma hypoxia is associated with the aggressiveness of neuroblastoma tumor independently from *MYCN *amplification.

## Discussion

Identification of new markers for outcome prediction will improve the effectiveness of risk related therapy for neuroblastoma patients. Furthermore, definition of the molecular programs linked to the prognostic markers is important for targeted therapy and specific drug discovery. Utilizing an innovative biology-driven approach, we selected 62 probesets representing a robust hypoxia signature of neuroblastoma cell lines and we tested it on a cohort of 88 neuroblastoma patients for outcome prediction. We found that NB-hypo is a strong, independent risk predictor also for patients with *MYCN *not amplified tumors. These results demonstrate the power of the biology-driven approach to identify molecular programs related to tumor progression and point to hypoxia and NB-hypo as important prognostic indicators in neuroblastoma.

The precise definition of the relationship between hypoxia and human cancer is complicated by the difficulty in measuring hypoxia at the tumor site. The use of an independent hypoxia signature to analyze tumor gene expression profile and related outcome is a powerful way to probe the hypoxic status of tumors and its implications [30,35]. We previously identified an 11 probesets hypoxia signature in 9 neuroblastoma cell lines by using *l*_1 _- *l*_2 _feature selection technique, a multivariate method specifically adopted to detect the subtle hypoxia response masked by strong transcriptional pattern [28]. The present study is based on the analysis of 11 neuroblastoma cell lines and on the modification of the *l*_1 _- *l*_2 _algorithm parameters in order to consider additional discriminative features. Furthermore, we combined the unique discriminating power of* l*_1_-*l*_2 _algorithm with the biological weight of the differential expression analysis to develop a robust method to identify a hypoxia signature that could better account for the biology of the tumor. This framework was carefully tested in its components and it is based on rigorous statistics and verification.

The definition of NB-hypo as a risk factor was obtained by estimating the Kaplan-Meier survival curves and the Cox proportional hazard regression model. We found that the NB-hypo stratifies neuroblastoma patients into good and poor prognosis groups with a significant separation of the patients for both OS and EFS. Risk assessment in neuroblastoma is based upon a number of factors which include, among others, age at diagnosis, International Neuroblastoma Staging System (INSS) stage, and *MYCN *status [36]. However, despite elaborate risk estimation strategies, outcome prediction for patients with neuroblastoma is still imperfect, as suggested particularly by current low- and intermediate risk patients with adverse outcome [34]. Our finding that NB-hypo is a true independent prognostic factor may help improving the risk assessment in neuroblastoma patients.

We and other have observed that the response to hypoxia is highly heterogeneous in different cell lines [28,35] raising the question of the effectiveness of the hypoxia signature generated in other cell types in stratifying neuroblastoma tumors. We found that the hypoxia gene set derived from dendritic cells did not stratify the patients. Furthermore, the single *VEGF *gene did not divide the patients in significant risk related groups even if it is almost universally induced by hypoxia, indicating that a more complex gene set was needed. Furthermore, we studied the relationship between NB-hypo and other hypoxia signatures generated in different tumor systems in terms of overlapping, neuroblastoma patients' stratification and risk assessment. We found that only a limited overlapping consistent with the notion that hypoxia modulates different genes in different cells. Furthermore, only the Winter's hypoxia signature [30] out of the 5 tested was able to stratify neuroblastoma patients but without reaching a level of significance in the multivariate Cox analysis. These results demonstrated that NB-hypo is the only independent risk factor, among the signatures tested, capable of producing a significant patients' stratification. This property of NB-hypo could be due to the match between cellular system used to derive the signature and tumor, to the rigorous computational framework utilized or, more likely, to a combination of these factors.

NB-hypo probesets are highly expressed in aggressive tumors indicating that neuroblastoma hypoxia leads to growth, metastasis and poor outcome when reaching the levels that make it measurable by microarray. These results provide evidence that hypoxia is a sizable component of progressing neuroblastoma tumor. Hypoxia is a common characteristic of many aggressive tumors [37] and there are several reports associating HIF-1α/2α expression with the patients' outcome in a broad range of cancers [38]. There is evidence that hypoxia plays a role in causing the dedifferentiation of neuroblastoma cells in vitro [39,40]. Furthermore, Forristal *et al*. [41] reported that HIF-2α is important in maintaining the pluripotency of human embryonic stem cells in hypoxic condition and Pietras *et al*. [42] demonstrated that HIF-2α maintains bone marrow- derived neuroblastoma tumor cells at a neural crest-like stage of differentiation in vitro and in vivo. Thus, the hypoxia-HIF-2α system promotes the undifferentiated phenotype either by dedifferentiation or inhibition of differentiation and may contribute to the aggressiveness through these mechanisms. The relationship between HIFs system and hypoxia is complicated by the fact that different environmental signals, such as genetic alterations, transition metals, chelating agents, hormones, and growth factors, share with hypoxia the property of inducing HIF-1α/2α and HIF-dependent gene transcription under normal pO_2 _[38,43,45]. Several reports associate HIF expression with the outcome of a broad range of cancers [38]. Correlation between HIF-2α, VEGF expression and poor prognosis [23] or pro-angiogenic activity was reported in neuroblastoma [46]. Recently, Noguera *et al*. [47] demonstrated an independence between HIF-1α and HIF-2α expression in neuroblastoma specimens and a correlation between HIF-1α and favorable outcome. These results show that the expression of HIF-1α/2α is not a specific and universal indicator of tissue hypoxia and the prognostic significance of HIFs may be unrelated to the hypoxic status of the neuroblastoma tumor. Thus, the assessment of tissue hypoxia requires measurement of multiple markers and NB-hypo could be a relevant tool for this purpose.

A computational approach using a priori biological knowledge to analyze the data of clinical neuroblastoma studies was applied successfully to the analysis of MYCN transcriptional targets whose over-expression contributes to the prediction of relapses and death from neuroblastoma [48]. The importance of MYCN in risk assessment is shown by the fact that *MYCN *gene is amplified, and often over-expressed, in about 22% of all neuroblastoma patients and it is an independent predictor for poor prognosis [49]. All metastatic tumors with amplified *MYCN *genes are aggressive, whereas metastatic tumors with non amplified *MYCN *genes have variable clinical behaviors influenced by the patient's age at diagnosis [50]. A 55-gene signature derived from the expression profile of metastatic neuroblastoma lacking *MYCN *amplification provided a new definition of high and low risk of disease progression [51]. We pursued the study of *MYCN *not amplified patients and tested the NB-hypo for its ability to predict the outcome of the disease. We analyzed the 72 *MYCN *not amplified patients of our cohort and we found that the NB-hypo significantly stratified them into good and poor prognosis groups and it was an independent risk factor relative to age and INSS.

Among *MYCN *not amplified tumors, stage 4 age>1 year patients are classified as high risk whereas the remaining are classified as low or intermediate risk based, among other factors, on age at the diagnosis and stage. We observed situations in which NB-hypo distinguished the clinical outcome of *MYCN *not amplified tumors more accurately than age or stage. In fact, NB-hypo correctly classified not only the deceased stage 4 age>1 year patients in the poor prognosis group, but included also in this category the deceased two stage 3 and the one stage 2 patients of the cohort belonging to the intermediate and low risk categories respectively. These results point to a high resolution of our signature. However, validation in larger cohort is needed to asses the effectiveness of NB-hypo in the above mentioned groups of patients. Although *MYCN *amplification is associated to the progression of the disease [5,8], our results indicate that hypoxia is related to the aggressiveness of neuroblastoma independently from *MYCN *amplification and it might be responsible for increasing the risk of poor outcome in patients with a more favorable biology.

Several microarray studies on neuroblastoma gene expression were published using different platforms and analysis methods [51-55]. Platform heterogeneity complicates the comparison of the signatures, although useful information can be obtained also from these datasets by appropriate algorithms [56]. The analysis of neuroblastoma tumor gene expression profiles, pioneered by Wei *et al*. [52], was followed by studies confirming the forecast of individual survival from gene expression data analyzed by the CASPAR algorithm [57,58]. Differences in the specific pattern of gene expression were described in spontaneous regressing, progressing [59] and intermediate risk [54] neuroblastoma. However, the overlap in identified expressed genes with prognostic information is low. Recent work by Vermeulen *et al*. [60] showed that a 59-gene signature, originated from the re-analysis of published gene expression studies, is an accurate and robust predictor of neuroblastoma outcome. These gene expression-based predictors were derived from the supervised association between the gene expression profile and clinical outcome. One shortcoming of this approach is that it is difficult to relate the signatures to the pathophysiology of the tumor; the second is that the results must be validated on an independent cohort of patients different from that from which the signature was originated. One advantage of the biology-driven approach is the immediate appreciation of the molecular program related to the prognostic indication. The second advantage is that independent validation of the results is not needed because the signature is not derived from the tumor profiles. Utilizing a biology-driven approach Chi *et al*. [35] demonstrated the prognostic value of hypoxia gene signature in breast and ovarian cancer. These results, together with our findings, show the power of controlled ex vivo studies in defining hypoxia as critical molecular programs in cancers and the potential for improvement in the current criteria for risk stratification of cancer patients.

The characterization of the tumor at diagnosis is indispensable for deciding the treatment and includes the evaluation of risk factors such as *MYCN *amplification status, hystotype, tissue markers, and chromosomal rearrangements [36]. The gene expression signatures are novel tools that may improve the stratification of patients thereby conditioning the choice of treatment. The hypoxic status detected by NB-hypo may be important to identify the tumors that may have high genetic instability [61] and high content of undifferentiated cells [25] at the time of excision as result of hypoxic phenotype. These characteristics of the primary tumor may be those that promote the aggressiveness of the disease and could be targeted by individualized therapies. There is a keen interest in biomarkers contained in body fluids because they are easy obtainable and can give indications about the response to therapy. These biomarkers includes catecholamines, ferritin, LDH [62], and other factors such as midkine [63], RANKL and OPG [64] but they do not define all risk groups and they can not substitute the analysis of the tumor mass. For the time being, the combination of tumor and body fluid characterization is needed for the optimal assessment of the treatment. It is reasonable to foresee that the study of the tumor mass will lead to the choice of individualized therapy and the longitudinal assessment of body fluids' biomarkers will monitor the response to therapy.

The prognostic molecular signatures linked to in vitro experimental models provide a more direct route to the development of targeted therapeutics. Many therapeutic agents are already under development to specifically target HIF pathways [65,66] or to target cells under hypoxic environments by hypoxia dependent gene therapy [17]. An alternative approach is to target the proangiogenic factors induced by hypoxia such as VEGF and its receptors [67]. The clinical results obtained so far are not very promising (reviewed in [68]) because anti-VEGF therapy causes vascular regression, with concomitant increase of intratumor hypoxia, activation of HIF target genes and increases in aggressiveness and metastatic spread [69,70]. These results suggest that the hypoxia, rather than of angiogenesis, may be the critical target for neuroblastoma therapy.

## Conclusions

Neuroblastoma shows notable heterogeneity with regard to histology and clinical behavior, ranging from rapid progression and poor clinical outcome to spontaneous or therapy-induced regression. Despite elaborate risk estimation strategies, outcome prediction for patients with neuroblastoma is still imperfect and requires new molecular indicators. We utilized an innovative biology driven approach to study the prognostic power of the molecular signature of hypoxia, a condition of low oxygen tension developing in the tumor mass. We demonstrated that the hypoxia signature is a strong, independent risk predictor in neuroblastoma patients. Our signature will improve the effectiveness of risk related therapy by helping patients' stratification. Furthermore, our result point to the transcriptional response to hypoxia as a negative event leading to poor outcome, suggesting that those neuroblastoma patients classified as high risk by our signature may benefit from therapeutic protocols targeting hypoxia pathway.

## Competing interests

The authors declare that they have no competing interests.

## Authors' contributions

PF and LV conceived the initial idea, the experimental design, supervised the work, and wrote the manuscript. PF performed microarray experiments and interpreted results. AB contributed with the development of MATLAB and R scripts for data processing, normalization and analysis, performed the supervised analysis, and visualized the results. SM wrote the core code for *l*_1 _- *l*_2 _regularization and contributed with the development of MATLAB and R scripts for data processing, normalization and analysis. LR performed the unsupervised analysis and helped in the design and implementation of the supervised analysis. AV supervised the entire statistical data analysis. RV organized tumor samples experiments and supervised the work. HC and JM organized tumor series collection. IØ collected all survival data, sectioned tumors for expression arraying and reviewed the tumor samples. MP performed hypoxia experiment on cell lines. AE interpreted the results and supervised the work. All authors read and approved the manuscript.
